# Proposing a strategy based on body-thermal status to improve the welfare of heat-stressed and water-deprived goats (*Capra hircus*)

**DOI:** 10.5713/ab.24.0096

**Published:** 2024-05-07

**Authors:** Emad M. Samara, Mohammed A. Al-Badwi, Khalid A. Abdoun, Ahmed A. Al-Haidary

**Affiliations:** 1 Department of Animal Production, College of Food and Agriculture Sciences, King Saud University, P.O. Box 2460, Riyadh, 11451, Saudi Arabia

**Keywords:** Adaptive Heterothermia, Body Core Temperature, Circadian Rhythm, Food Security, Thermoregulation, Water Restriction

## Abstract

**Objective:**

Despite the considerable body of research on the effects of heat stress coupled with water scarcity (either through restriction or deprivation) on goats, aimed at enhancing their welfare, there remains a notable gap in the literature regarding the subsequent period following water restoration, during which the cumulative impact is fully alleviated. In response to this gap, we propose a strategy grounded in the assessment of body-thermal status to improve the welfare of heat-stressed and water-deprived goats. Specifically, our strategy seeks to determine the minimally required recovery interval necessary to completely mitigate the residual effects of water deprivation endured for a duration of 72 hours.

**Methods:**

Eight healthy Aardi bucks, aged 10 months and weighing 30 kg, were subjected to three distinct stages: euhydration, dehydration, and rehydration. Each stage spanned for 72 hours except for the rehydration stage, which was left unrestricted. Various meteorological, biophysiological, and thermophysiological measurements were subsequently recorded.

**Results:**

Exposure of heat-stressed goats, as indicated by the temperature-humidity index values, to a 72 hours deprivation period resulted in noticeable (p<0.05) alterations in their biophysiological (daily feed intake, body weight, and feces water content) and thermophysiological responses (core, rectal, skin, and surface temperatures, respiratory and heart rates, internal, external, and total body-thermal gradients, heat tolerance and adaptability coefficients, heterothermial total body-heat storage, and total water conservation). Remarkably, our findings demonstrate that all assessed variables, whether measured or estimated, returned to their baseline euhydration levels within 10 days of commencing the rehydration phase.

**Conclusion:**

In order to improve the welfare of heat-stressed and 72 hours water-deprived goats, it is imperative to allow a recovery period of no less than 10 days following the restoration of water access prior to initiating any subsequent experiments involving these animals. Such experiments, addressing these critical aspects, serve to advance our understanding of goat welfare and obviously hold promise for contributing to future food security and economic viability.

## INTRODUCTION

Among the pivotal determinants shaping contemporary socio-economic paradigms, significant attention is drawn to the dynamics of climate change projection, escalating human population growth, and the imperative of ensuring food security [1–3]. In comparison to other ruminants and livestock species, goats stand out as an ideal climate-resilient animal model, endowed with exceptional morphophysiological, thermophysiological, and behavioral attributes that enable them to adeptly navigate diverse stressors and thrive in challenging environments [4–7]. As the epitome of domesticated animals well-suited for adverse conditions, goats assume paramount importance in mitigating the impacts of climate change. Indeed, owing to their low-input, high-output production systems, goats emerge as the preferred future player in ensuring sustainable and profitable livestock production, thereby optimizing economic returns either by the large-scale enterprises or among the marginal small-scale farmers [8,9].

Despite such exceptional plasticity and adaptive capacities, the productivity of goats can be compromised under specific environmental circumstances. For instance, periods of water scarcity, whether through restriction or deprivation, could impact their wellbeing. Indeed, withholding water for extended durations has been shown to elevate goats’ body temperatures, respiratory and heart rates (HR), and intensify the occurrence and duration of selective brain cooling phenomena [6,7,10]. Furthermore, dehydrated goats exhibit reduced feed intake (FI), feces moisture content, urine output, and blood concentrations of thermogenic hormones [11,12]. Consequently, subjecting goats to complete or partial water restriction in conjunction with heightened environmental temperatures can trigger substantial deviations in their homeostatic and homeokinetic physiological responses, prompting the activation of various mechanisms to cope with such conditions. Typically, goats under such circumstances tend to minimize water loss and thermogenic mechanisms while enhancing water conservation and thermolytic mechanisms. These collective responses manifest in noticeable impacts at both organ and cellular levels, detrimentally affecting meat, milk, and wool production, and consequently compromising the welfare and wellbeing of goats [3,13,14]. Consequently, the assessment of goat welfare across all production phases, employing animal-based or management-based approaches that encompass morphophysiological, thermophysiological, behavioral, and productive responses, alongside considerations of housing conditions, health plans, and resource management, is imperative for ensuring food security and optimizing economic returns [9].

Obviously, it is quite evident that extensive research has been conducted on heat and water restriction stresses in ruminants, particularly in goats, aimed at gaining a comprehensive understanding of the physiological mechanisms underlying their thermal and drought tolerance, and ultimately, enhancing their welfare and overall resilience. However, despite the breadth of research on these stressors, the existing body of knowledge does not provide insight into the duration of water restoration required for the physiological system of heat-stressed goats to fully alleviate the carryover effects of water deprivation. Investigations focusing on these aspects would contribute significantly to our understanding of goat physiology and undoubtedly contribute to enhancing their welfare amidst an environment undergoing change. Therefore, based on observations of changes in body thermal status in heat-stressed goats subjected to dehydration (DE) followed by a prolonged period of rehydration, we propose a simplified strategy to improve their welfare through determining the minimum required recovery interval necessary to eliminate the carryover effects of both stressors before conducting any experiments on them in the future.

## MATERIALS AND METHODS

The current experiment was conducted in adherence to the ethical guidelines established by the Institutional Research Committee of King Saud University, Riyadh, Saudi Arabia (protocol number: KSU-SE-21-84), which rigorously upholds standards for the welfare and ethical treatment of animals employed in scientific inquiry.

### Animals, management, and experimental design

During the summer season, a cohort comprising eight healthy bucks of the native Aardi breed, characterized by a black and white coat coloration, and exhibiting an average body weight (BW) range of 25 to 30 kg and an age range of 10 to 12 months, were individually housed throughout the experimental period within shaded pens measuring 1.50×1.50 m. These bucks were provided with a commercial complete Al-Wafi pelleted diet (manufactured by Arabian Agricultural Services Co., Riyadh, Saudi Arabia) twice daily at 7:00 h and 16:00 h, with feed allocation set at 3% of their respective BWs, as per previously outlined dietary compositions and specifications [15]. Additionally, the animals had continuous access to water *ad libitum*, except during the DE stage, and were afforded free access to mineral blocks. Moreover, consistent medical care, including deworming, vaccination and routine inspection, was administered to all bucks throughout the duration of the experiment.

The entire experimental procedure was structured into two distinct phases. During the preparatory phase, spanning approximately 19 days, the bucks underwent surgical implantation of transmitters to telemetrically record their body-core temperature (Tc). Following the surgical procedure, the animals were carefully monitored during the healing process and acclimatized to the experimental environment. Additionally, they were gradually introduced to the feeding regimen and familiarized with the measuring equipment. During the experimental phase, lasting around 16 days, on the other hand, bucks were subjected to three distinct treatments/stages: euhydration (EU), DE, and rehydration (RE). Each stage endured for a duration of 72 hours, except for the RE stage, which was left unrestricted. Throughout the experimental phase, various meteorological, biophysiological, and thermophysiological data were meticulously collected. Notably, no apparent discomfort was observed among the bucks during the DE stage. More importantly, measurements were conducted on the 4th, 6th, and 8th day of the RE stage, following the initial 72 hours. However, it was observed that the residual effects of water deprivation persisted across all measured variables until the 10th day after water restoration. Consequently, the experiment was promptly concluded, and data recorded on this day (referred to as RE10) were exclusively presented herein.

### Experimental measurements

Dry-bulb ambient temperature (Ta) and relative humidity (RH) were continuously monitored at 10-minute intervals using four high-precision HOBO H-08 Pro Series data loggers (manufactured by Onset, Bourne, MA, USA), strategically positioned inside the pens and above the animals at an approximate height of 2 m from the ground. The accompanying Box-Car Pro 4 software (Onset, USA) was utilized for the programming of these loggers and subsequent data retrieval. To assess the environmental conditions experienced by the experimental bucks, the recorded Ta and RH data were utilized to compute the temperature-humidity index (THI) employing a formula adapted from Kelly and Bond [16]. The resulting circadian rhythms were analyzed to determine key parameters including mesor (representing the mean-level or mid-line statistic of the rhythm), zenith (indicating the maximum value of the rhythm), acrophase (denoting the time corresponding to the rhythm’s zenith), nadir (representing the minimum value of the rhythm), trough (indicating the time of the rhythm’s nadir), and the thermal load of each stage (calculated as the difference between the rhythm’s zenith and nadir values).

With the exception of the DE stage, the daily water intake (WI) of the experimental bucks was assessed each morning at 08:00, prior to the provision of fresh meals, by measuring the remaining water from the previous day. Water evaporative loss was quantified by placing 6 L of water in an empty bucket to mimic the prevailing ambient conditions, and the corrected WI was subsequently calculated. Notably, during the RE stage, the WI was also measured within the first 30 minutes to ascertain the immediate water consumption by each buck following water restoration. Concurrently, the daily FI was monitored throughout the experiment by weighing the remaining feed from the previous day using a single-pan balance accurate to the nearest 20 g. Furthermore, the bucks’ BW was determined using a standard balance precise to the nearest 100 g. To assess the percentage of BW replenishment after rehydration, BW was recorded 30 minutes after the commencement of the RE stage. Additionally, feces samples were collected daily to determine their water content (FWC) through oven drying at 105°C for a duration of 6 hours.

A CorTemp system (HQ Inc., Palmetto, FL, USA) comprising precalibrated telemetric-temperature transmitters, which were surgically implanted intraperitoneally following sterilization, along with data recorders housed within custom-designed pouches affixed to the animals’ backs, was utilized for the recording of the circadian rhythm of the bucks’ Tc at 10-minute intervals. The perioperative surgical procedure, encompassing preoperative, intraoperative, and postoperative phases, was conducted in accordance with the previously outlined protocol by Samara et al [17]. Subsequent to data collection, similar to the analysis conducted on meteorological data, the recorded Tc data were subjected to analysis to determine key circadian rhythm parameters, including mesor, zenith, acrophase, nadir, and trough, as well as the rhythm’s range of oscillation, calculated as the difference between the rhythm’s zenith and nadir values, over a 24-hour period.

**Figure 1:**
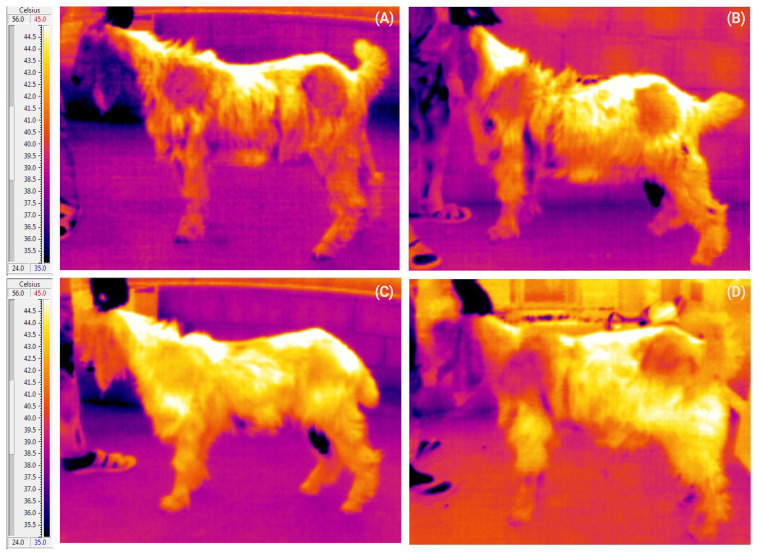
Representations of full-body thermograms of a buck following 72 hours of the euhydration stage (A), 72 hours of the dehydration stage (B), 72 hours of the rehydration stage (C), and after 10 days of the rehydration stage (D). A rainbow color palette was employed for all thermograms for enhanced visual contrast and interpretation.

Rectal (Tr), skin (Tsk), and surface (Ts) temperatures, alongside respiratory (RR) and heart rates (HR), were all recorded once daily at 12:00 throughout the duration of the experiment. Rectal temperature (Tr) of the bucks was measured employing a calibrated digital rectal thermometer, featuring a temperature range of 37.5°C to 43.0°C and a resolution of 0.10°C. Skin temperature (Tsk) in two regions of daily shaved skin, each 2×2 cm in size, located at the right shoulder and hip, was assessed using a Traceable Mini-IR infrared thermometer (manufactured by Friendswood, TX, USA), capable of measuring temperatures within a range of −22°C to 110°C, with a resolution of 0.10°C and an accuracy of ±1.00°C (between 15°C and 40.0°C). Additionally, a portable forward-looking and automatically calibrated VisIR-Ti200 infrared camera (manufactured by Thermoteknix Systems Ltd., Cambridge, UK) equipped with 25° lens, 1.3 M pixel visible camera, LCD touch screen, a 7.5 to 13 μm spectral range, and a precision of ±0.1°C was placed perpendicular and approximately 100 cm away from buck’s surface to obtain both sides’ thermograms (infrared thermographic images) of their whole body in duplicate. To ensure accurate surface temperature (Ts) measurements, parameters such as the emissivity of the animal surface, surrounding temperature, and distance between the animal and the camera were supplied to the camera prior to capturing. A total of 130 thermograms were obtained and analyzed using the accompanying software, TherMonitor (manufactured by Thermoteknix Systems ltd, UK), to determine the maximum Ts in the regions of interest. Figure 1 illustrates some examples of full-body thermograms of a buck undergoing various experimental stages. Concurrently, RR and HR were assessed using a 3M Littmann stethoscope, positioned between the 9th and 11th, and 3rd and 6th intercostal spaces, respectively. By counting 10 breaths and beats, the recorded time was expressed as the number of breaths/beats per minute to determine RR and HR, respectively.

Several formulas, including [Tc-Tsk], [Tsk-Ta], and [Tc-Ta], were used in accordance with da Silva and Maia [18] to compute internal (iBTG), external (eBTG), and total (tBTG) body-thermal gradients, respectively, utilizing data collected at 12:00. The heat tolerance coefficient (HTC) of the experimental bucks was determined utilizing Rhoad’s Iberia heat tolerance test [19], while their adaptability coefficient (AC) was calculated following the method outlined by Martins Júnior et al [20]. Furthermore, bucks’ heterothermial total body heat storage (tBHS) and water conservation (WC) were evaluated for each experimental stage employing the methodology proposed by Ostrowski et al [21].

### Statistical analysis

The acquired data underwent analysis using the statistical analysis system (SAS Institute v9.4, Inc., Cary, NC, USA), while graphical representations were generated utilizing SigmaPlot software (SigmaPlot v14.0; Systat Software Inc., San Jose, CA, USA). Descriptive statistics were computed using the PROC MEANS procedure. To compare differences in variables before and after DE, the PROC TTEST procedure was employed, utilizing a pretest/posttest design wherein the bucks were evaluated before DE (pretest) and after RE (posttest). Paired two-tailed Student’s t-tests, assuming equal variances, were applied to assess pairs of data, with statistical significance set at p<0.05. Unless otherwise specified, means and their pooled standard error of the mean were reported.

## RESULTS

### Meteorology

**Figure 2:**
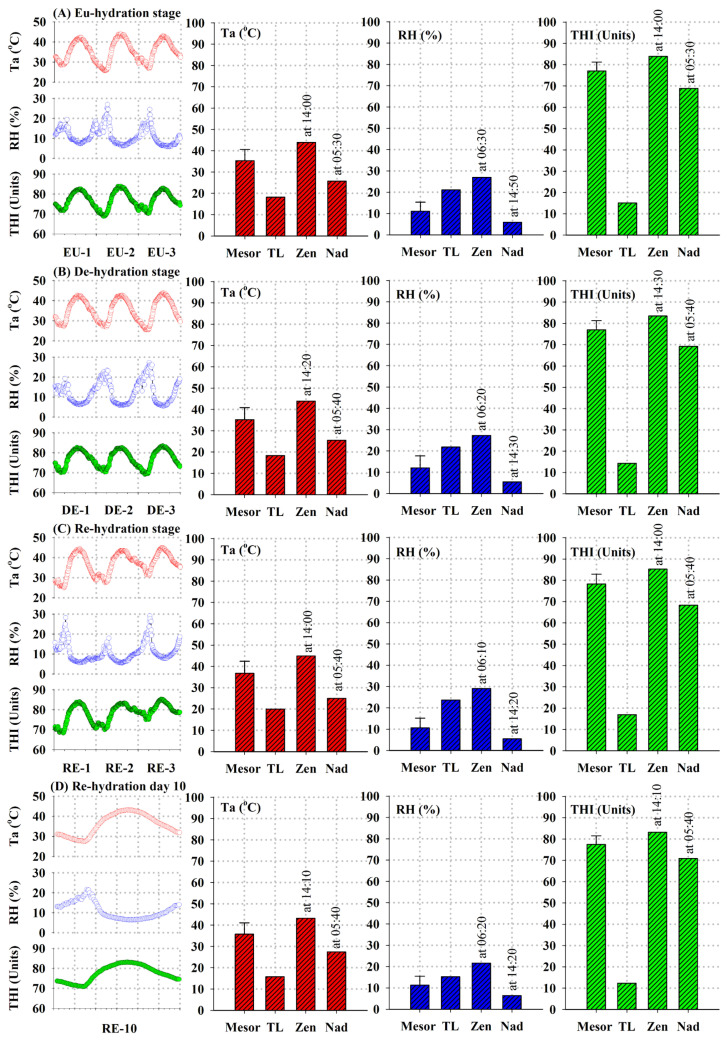
Meteorological data, including ambient temperature (Ta) in °C, relative humidity (RH) in %, and the temperature-humidity index (THI) in standardized units, were monitored across distinct phases of the current experiment. The experimental stages are denoted as EU-1 to 3 (euhydration), DE-1 to 3 (dehydration), RE-1 to 3 (rehydration), and RE-10 (day 10 of rehydration). Line charts are utilized to illustrate the daily circadian patterns observed at 10-minute intervals, while vertical bar charts are employed to portray the rhythms’ mesors (mean values±standard deviation), thermal loads (TL), zeniths (Zen), acrophases, nadirs (Nad), and troughs (refer to the accompanying text for further elucidation).

**Figure 3:**
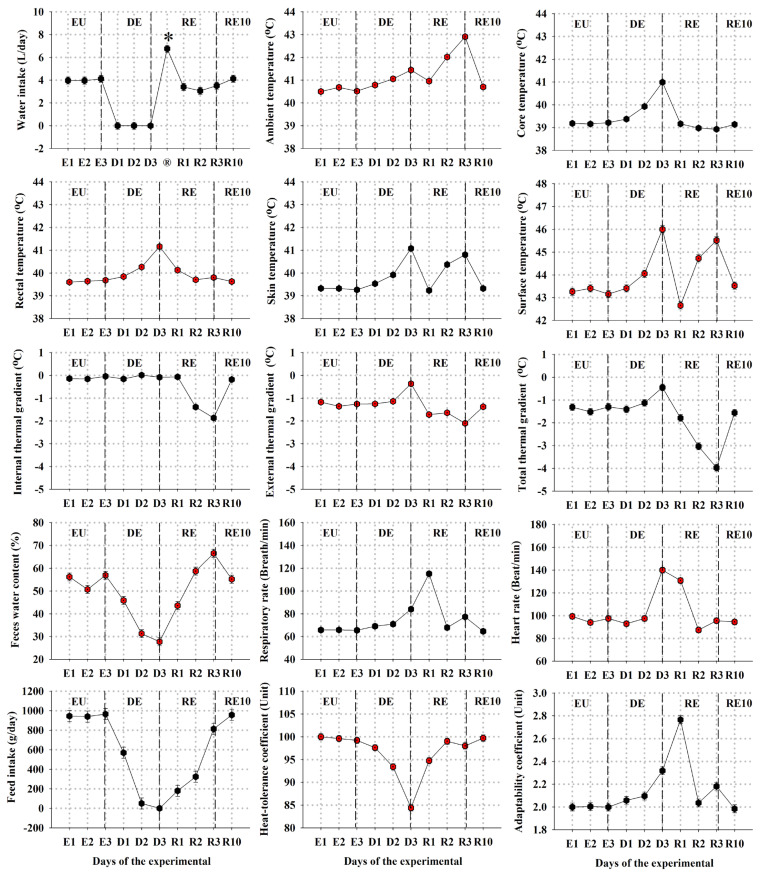
Alterations in daily averages of select meteorological, biophysiological, and thermophysiological parameters recorded/estimated across distinct phases of the current experiment (mean±standard error). The experiment includes euhydration (EU), dehydration (DE), rehydration (RE), and day 10 of rehydration (RE_10_). It is imperative to note that the biophysiological metrics were assessed at 08:00 hours, whereas the meteorological and thermophysiological data were measured/estimated at 12:00 hours. The asterisk (*) denotes the volume of water ingested promptly following 30 minutes of water reinstatement (®).

Meteorological parameters collected during various experimental stages are depicted in Figure 2. The recorded Ta, RH, and THI data exhibited monophasic daily rhythms, with nadir values of Ta and THI observed during the early morning hours (05:00 to 07:00 h) and zenith values recorded in the afternoon (13:00 to 15:00 h), whereas RH displayed an inverse trend (Figure 2). With the exception of the 2nd and 3rd days of the RE stage (Figure 3), no discernible differences in the circadian rhythms of these parameters were observed across the experimental days. Indeed, the consistent values of calculated Ta, RH, and THI parameters, including mesor, zenith, acrophase, nadir, trough, and thermal loads for the stages, suggest a reasonable approximation of uniform environmental conditions throughout the experiment (Figure 2). Notably, based on the calculated average THI, the bucks appeared to be experiencing heat stress throughout the experimental period [18–22].

### Biophysiology

Water deprivation for 72 hours resulted in a statistically (p<0.05) decrease in the overall means of bucks’ FI, BW, and FWC beginning from the first day of water deprivation (Table 1; Figure 3). The corresponding reductions of these variables during the DE stage were approximately 80%, 20%, and 36% compared to the EU values. Upon regaining access to water in the RE stage, the bucks consumed an average of 6.75±0.30 L of water during the initial 30 minutes of water restoration (Figure 3). Notably, with the exception of WI and FI, bucks’ BW and FWC were restored following RE until the conclusion of the experiment (Table 1). However, all these variables returned completely to their EU levels (p>0.05) after 10 days from the initiation of the RE stage (Table 1; Figure 3).

### Thermophysiology

**Figure 4:**
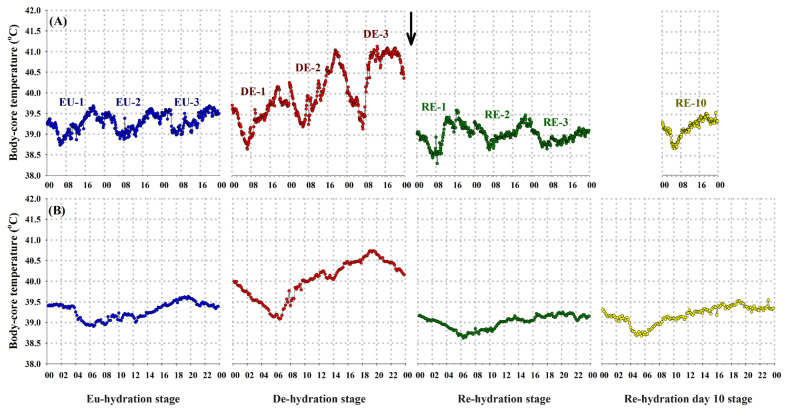
The recorded circadian rhythms of body-core temperature (measured in °C) at 10-minute intervals in heat-stressed and water-deprived buck goats across consecutive experimental phases (A) are presented alongside their respective aggregate averages (B). The experimental stages include euhydration (EU), dehydration (DE), rehydration (RE), and day 10 of rehydration (RE_10_). Arrow indicates when water was restored.

During the EU stage, the recorded results indicated that the bucks’ Tc, captured at 10-minute intervals, exhibited a monophasic circadian rhythm resembling the pattern observed in the recorded Ta rhythm. However, the nadir values of Tc were noted during the early morning hours (05:30 to 06:30 h), with zenith values observed at the end of the day (19:00 to 20:00 h), and a range of oscillation of approximately 0.72°C (Table 2; Figure 4). The combined effect of heat stress and water deprivation had impacted the recorded Tc. In fact, the overall mean of Tc increased (p<0.05) from 39.18°C± 0.02°C during the EU stage to 40.09°C±0.02°C during the DE stage, with the rhythm’s range of oscillation increasing to 1.65°C (Table 2). Notably, this influence was initially imperceptible within the first 24 hours following water deprivation but became evident thereafter (Figure 4). For instance, a range of oscillation of up to 2.00°C and a backward shift in the acrophase of Tc zenith values (reaching as high as 41.15°C) occurring during the midday hours (12:00 to 13:00 h) were observed after 72 hours of water deprivation (Table 2). However, upon rehydration, these observed changes gradually began to revert to their EU levels. The overall mean of bucks’ Tc (p<0.05) decreased to 39.01°C±0.02°C, and the rhythm’s range of oscillation also decreased to 0.63°C. Additionally, a distinct forward shift in the acrophase of Tc zenith values was observed, occurring initially at 16:00 to 17:00 h after 24 hours, and further shifting to 21:00 to 22:00 h after 48 hours of water restoration (Table 2). Ultimately, all these changes returned to EU levels (p>0.05) after 10 days of RE (Table 2; Figure 4).

Moreover, the overall means of Tr, Tsk, Ts, RR, HR, eBTG, tBTG, and AC demonstrated a (p<0.05) increase, while the overall mean of HTC (p<0.05) decreased due to the imposed water deprivation (Table 1; Figure 3). Surprisingly, however, the overall mean of iBTG remained unchanged (p>0.05) throughout the DE stage. Following water restoration, the overall means of several variables began to return to their EU levels, except for iBTG, which (p<0.05) decreased, and the overall means RR and AC continued to (p<0.05) increase (Table 1). Once again, all variables completely returned to their EU levels (p>0.05) 10 days after initiating the RE stage (Table 1; Figure 3). Above all, the estimated average value of bucks’ tBHS and the corresponding estimations of WC (p< 0.05) increased by 209% during the DE stage, then (p<0.05) decreased to 135% during the RE stage, and returned back to their EU levels (p>0.05) after 10 days of RE (Table 1).

## DISCUSSION

For small ruminants, as for any living creature, water plays a crucial role as an essential nutrient for maintaining fluid and electrolyte balance, facilitating thermoregulation, and ensuring overall survival. These animals generally exhibit lower absolute daily water requirements (expressed as L/d) compared to larger ruminants [23–25]. Even when compared to similar body size and naturally adapted sheep, goats demonstrate lower absolute water turnover, ingesting approximately 30% less drinking water compared to similarly sized and naturally adapted sheep, owing to their superior water economy mechanisms [6–8]. Extensive literature and reviews have highlighted goats as remarkably efficient in enduring periods of water scarcity, lasting beyond three to four days [6,11,12,25,26]. However, when coupled with heat stress, this situation can significantly exacerbate stress levels. Indeed, the combination of heat stress and water scarcity is widely acknowledged as a potent stressor impacting the production performance of goats [27–29], thereby affecting future food security and economic returns. Consequently, enhancing the welfare of goats reared under such environmental conditions is of paramount importance. The current experiment was designed to investigate changes in body thermal status, encompassing both bio- and thermophysiology, in goats subjected to heat stress and concurrent 72-hour dehydration followed by a 10-day RE period. This exploration aimed to determine the minimally required recovery interval before conducting further experiments on these animals.

### Biophysiology

Based on the acquired findings, it is evident that subjecting heat-stressed goats to 72 hours of water deprivation induced noteworthy changes in their biophysiological responses (Table 1; Figure 3). In fact, these alterations commenced promptly, with a marked reduction in FI observed as early as 24 hours into the DE stage, reaching approximately 45% of the EU value. This reduction progressively intensified, reaching approximately 95% of the EU value by the end of the DE stage. This phenomenon can be elucidated by the well-established interrelation between WI and FI in ruminants and other mammals, arising from the close association between water turnover and energy production [13,14,30]. The progression of dehydration in ruminants typically unfolds in two phases [31]. Phase I is characterized by sustained FI and salivation to facilitate normal fermentation in the foregut, while phase II witnesses a severe decline in both FI, salivation, and rumen content. Although our observations align with earlier reports on Aardi goats [11,26], the observed decrease in FI in this study surpassed previous findings, suggesting a swifter transition into phase II of dehydration. Conversely, Hassan [32] documented that Egyptian Baladi goats maintained relatively high feeding levels (35% of their EU level) for three days of water deprivation, while Bedouin goats continued feeding for six days [24]. Discrepancies in these findings may be attributed to the prevailing high Ta during our experiment, as reductions in FI during water deprivation tend to be more pronounced under elevated Ta [33]. Notwithstanding such evidences, the rapid reduction in FI observed from the onset of the DE stage can be attributed to the pellet-based feeding regimen employed, representing an adaptive thermoregulatory advantage aimed at reducing the heat increment from FI, lowering energy metabolism, and conserving body water [8]. Thus, adaptation to water scarcity appears to hinge on the efficient utilization of body water. Silanikove [13,14] proposed that drought tolerance is primarily gauged by the rate of BW changes, as any decrease in BW is intricately linked to reductions in FI and energy metabolism. In our experiment, water deprivation for 72 hours resulted in a modest 20% decrease in goat BW (Table 1). Compared to most monogastric mammals, Silanikove [4] concluded that the rumen serves as the primary source of systemic water during water deprivation, potentially explaining goats’ ability to endure up to 20% BW loss during dehydration. In agreement, El-Nouty et al [26] observed an 18% BW loss in Aardi goats after four days of dehydration during the summer season, while Indian desert goats, subjected to an intermittent watering regime during the summer, gained weight by season’s end [34], suggesting an inherent water-conserving mechanism. These findings accord as well with those reported by others on goats [11,35]. Another crucial mechanism employed by goats to conserve body water is feces desiccation [8,9,13]. Our findings revealed a 36% decline in FWC during the DE stage, consistent with prior research [11,26].

Following water restoration, bucks swiftly regained their BW, while their WI, FI, and FWC gradually returned to pre-dehydration levels (Table 1; Figure 3). Goats are known to rapidly replenish their water deficit in a single drinking session and can store up to 15% or more of their BW as water in their rumen [13,14,36]. Furthermore, Alamer and Al-Hozab [37] observed a correlation between the amount of water consumed immediately post-deprivation and seasonal variation in sheep, with replenishment accounting for 9.9%, 13.8%, and 18.9% of dehydrated BW in winter, spring, and summer, respectively. Black Bedouin goats were reported to replenish all water losses within minutes [38], while Aardi goats replenished 91% of their BW losses shortly after RE [11,26], and Najdi sheep required 5 hours to replenish their entire BW losses [37]. The findings of our experiment are in consistent with these earlier studies on goats. During the initial 30 minutes of the RE stage, experimental goats consumed an average of 6.75±0.16 L of water, equivalent to approximately 22% of their dehydrated BW (Figure 3). Notably, they replenished 102% of their BW losses, exceeding their loss during the DE stage, suggesting that the Aardi breed exhibits rapid recovery. Remarkably, all recorded biophysiological variables returned to pre-dehydration levels after 10 days of the RE stage (Table 1; Figure 3). Based on these observations, we tentatively suggest that a recovery period of at least 10 days post-water restoration should be provided before conducting any future experiments on heat-stressed and water-deprived goats. However, do the thermophysiological variables collected or estimated during the current experiment further support this tentative conclusion?

### Thermophysiology

Elevations in goats’ Tc, Tr, Tsk, Ts, RR, HR, eBTG, tBTG, and AC, as well as a reduction in their HTC, were initially observed after 24 hours of commencing the DE stage (Figure 3). To gain deeper insights into how such conditions affect the thermoregulatory system of goats, we can examine it from a thermodynamic perspective by balancing heat input against heat output. As proposed by da Silva and Maia [18], the quantity of heat input depends on the body’s heat sources (metabolism, work/exercise, and environmental gain), while the quantity of heat output depends on the rates of sensible, latent, or a combination of these avenues. When a homeothermic body like goats experiences a net heat input, it warms up, whereas a net heat output leads to cooling down. Thereby, body temperature generally reflects body heat content [38]. Our observations after 24 hours of water deprivation collectively provide substantial evidence that the insulation of bucks’ bodies began to constrict (progressing toward their minimum levels), and subsequent heat dissipation was initiated, mainly through sensible avenues. Such status, coupled with the observed unchanged iBTG value (Figure 3), attests that these responses were primarily activated during this period to facilitate heat flux from different regions of goats’ bodies to the environment, thereby maintaining their body thermal homeokinesis, particularly their Tc.

After 72 hours of water deprivation, the recorded Tc, Tr, Tsk, Ts, HR, eBTG, and tBTG reached their peak values, while the HTC reached its nadir values, indicating that sensible heat-loss avenues had maximized (or even approached the point of adding heat to the body), and latent evaporative avenues must increase to correspond with the observed elevation of tBHS (Table 1). Small ruminants mainly rely on respiratory evaporative cooling as a latent heat-loss mechanism [14]. However, evidence from the present experiment indicated that the RR (and AC) of heat-stressed and water-deprived bucks had slightly increased after 72 hours of water deprivation. This confirms earlier studies reporting insignificant differences in RR between water-deprived and euhydrated goats [26,40]. Nevertheless, given the current experimental conditions, our findings suggest that the observed slight increase in RR could be attributed to the prevailing high Ta. Previous studies have demonstrated that the RR of small ruminants is influenced by environmental Ta rather than their body temperature [41,42]. Furthermore, in addition to triggering body water conservation mechanisms, these animals are known to suppress latent evaporation (and hence heat dissipation) under water deprivation, contributing to fluctuations in their body temperatures [11,37,43]. This was evident herein in the observed elevations of Tc, Tr, Tsk, Ts, eBTG, and tBTG, the reduction of HTC, and the unchanged iBTG. Indeed, the combined effect of heat stress and 72 hours of water deprivation induced a range of oscillation of 2.00°C and a backward shift in the acrophase of Tc zenith values reaching as high as 41.15°C, occurring around midday (12:00 to 13:00 h), compared to a range of oscillation of around 0.72°C and a zenith value around 39.62°C occurring at the end of the day (19:00 to 20:00 h) during the EU stage (Table 2). Such findings might suggest that this homeothermic species possesses another significant water economy mechanism, namely, the capability of being thermolabile. Indeed, several articles have categorized heterothermy as a key adaptation phenomenon for some ungulates living in arid conditions [7]. To reduce latent evaporative heat loss during hot diurnal periods, body heat is stored and body temperatures are elevated, and then during cold nocturnal periods, body heat is dissipated using sensible means, allowing body temperatures to fall. As previously noted, the heterothermial characteristic of tBHS and the consequent conserved water (WC) of goats during different experimental stages were estimated according to Ostrowski et al [21]. Notably, the use of such a mechanism by heat-stressed and water-deprived goats resulted in a 209% increase in tBHS from 90.06 to 188.36 kJ/d in the EU and DE stages, respectively. Dissipation of such amounts of heat through the latent evaporative avenue would require 78.35 mL H_2_O/d during the DE stage compared to 37.46 mL H_2_O/d during the EU stage (Table 1). This indicates that the observed elevations of goats’ Tc, Tr, Tsk, Ts, eBTG, and tBTG, and the reduction of HTC during the DE stage compared to the EU stage are essential adjustments to minimize their body water losses during water deprivation. On the other hand, it should be noted that Mitchell et al [44] suggested that any animal exhibiting the heterothermy phenomenon should meet five criteria, and buck goats administered under the current experimental conditions only met four out of these five, suggesting that goats might lack the evidence to exhibit such phenomena. Nevertheless, further research is required to validate our findings in free and unrestrained goats under their natural habitats to allow these animals to fully express their physiochemical and thermoregulatory adaptabilities.

Once these goats regained access to water in the RE stage, our findings revealed a gradual return of all measured variables to their EU levels (Table 1; Figure 3). However, the RR and AC continued to increase for 24 hours after rehydration, returning to their EU levels after 48 hours. These observations suggest that during this particular period, the experimental goats primarily relied on latent evaporative cooling to mitigate the accumulated effects induced by heat stress and water deprivation over the past 72 hours. This reliance was especially evident during the first 30 minutes of the RE stage, during which they replenished their water deficit to its EU level by consuming an average of 6.75±0.16 L of water. Additionally, recorded Tsk and Ts were observed to increase after 48 hours and further after 72 hours following their initial decrease after 24 hours, while body-thermal gradients (iBTG, eBTG, and tBTG) decreased progressively after 48 and 72 hours of rehydration. These alterations can be primarily attributed to the prevailing Ta. Physiologically, the increases in Tsk and Ts (and consequently iBTG, eBTG, and tBTG) can be attributed to either direct radiative heat gain from the external environment or blood redistribution from visceral organs to body extremities during the hottest hours of the day [18,39]. Analysis of the daily average changes during the RE stage (Figure 3) indicates that these variables were influenced by a sudden and unexpected increase in Ta recorded at 12:00 on the 2nd and 3rd days of this stage. Therefore, conducting future research under biometeorologically-simulated environments seems imperative. Nevertheless, all thermophysiological variables returned completely to their EU levels after 10 days of starting the RE stage (Table 1; Figure 3), confirming the earlier conclusions drawn from the biophysiological variables.

## CONCLUSION

This research article proposes a strategy grounded in the assessment of body-thermal status to improve the welfare of heat-stressed goats. Specifically, our strategy seeks to determine the minimally required recovery interval necessary to mitigate the residual effects of water deprivation endured for a duration of 72 hours. Despite the fact that subjecting heat-stressed goats to such period of dehydration elicited considerable alterations in their body thermal status, current findings demonstrate that all variables returned to their euhydration levels after 10 days of initiating the rehydration stage. Based on these observations, we recommend a recovery period of at least 10 days after water restoration to eliminate the carryover impact of water deprivation before conducting further experiments on any heat-stressed goats. These findings hold significance for both economic animal production and ethical animal care practices. Research endeavors focusing on such aspects can enhance our understanding of goat welfare under challenging environmental conditions, subsequently improving their economic productivity. It is recommended to validate the obtained results at the molecular level using techniques like functional genomics in future research, both in confined goats raised under biometeorologically-simulated environments and in free-roaming goats in their natural habitats.

## Figures and Tables

**Table 1:** The influence of dehydration followed by subsequent rehydration on specific biophysiological and thermophysiological variables in heat-stressed buck goats

Variables^1)^	Experimental stages^2)^				SEM

	EU	DE	RE	RE_10_	
Biophysiology					
Water intake (L/d)	4.01^a^	-	3.32^b^	4.12^a^	0.30
Feed intake (g/kg^0.75^/d)	79.14^a^	15.71^c^	37.16^b^	78.27^a^	1.67
Body weight (kg)	27.32^a^	21.80^b^	27.15^a^	27.53^a^	1.12
Feces water content (%)	54.58^a^	34.93^b^	56.26^a^	55.15^a^	1.74
Thermophysiology					
Body-rectal temperature (°C)	39.64^b^	40.42^a^	39.88^b^	39.63^b^	0.07
Body-skin temperature (°C)	39.30^b^	40.17^a^	40.13^a^	39.32^b^	0.10
Body-surface temperature (°C)	43.27^b^	44.49^a^	44.30^a^	43.53^b^	0.15
Respiratory rate (Breath/min)	65.66^c^	74.63^b^	86.75^a^	64.58^c^	2.19
Heart rate (Beat/min)	96.93^c^	110.12^a^	104.55^b^	94.41^c^	2.48
Internal body-thermal gradient (°C)	−0.12^a^	−0.08^a^	−1.11^b^	−0.18^a^	0.09
External body-thermal gradient (°C)	−1.26^b^	−0.92^a^	−1.82^c^	−1.38^b^	0.06
Total body-thermal gradient (°C)	−1.38^b^	−1.00^a^	−2.93^c^	−1.56^b^	0.14
Heat tolerance coefficient (Unit)	99.60^a^	91.80^b^	97.25^b^	99.75^a^	0.68
Adaptability coefficient (Unit)	1.99^c^	2.16^b^	2.33^a^	1.98^c^	0.03
Body-heat storage (kJ/d)	90.06	188.36	121.53	87.89	-
Water conservation (mL)	37.46	78.35	50.55	36.56	-

SEM, standard error of the mean.

1) Biophysiological variables were measured at 08:00 during each day of the current experiment, while thermophysiological variables were measured/estimated at 12:00 (see text for more details). We must point out as well that the overall averages of the whole experimental stages are presented herein.

2) EU, euhydration stage; DE, dehydration stage; RE, rehydration stage; RE_10_, day 10 of the rehydration stage.

a–c Means bearing different superscripts are significantly different at p<0.05.

**Table 2:** Comprehensive examination of the recorded circadian rhythm of body-core temperature at 10-minute intervals in heat-stressed buck goats subjected to euhydration (for a duration of 3 days), dehydration (for a period of 3 days), and subsequent rehydration (spanning 10 days)

Parameters^1)^	Experimental stages^2)^										RE_10_

	EU			DE			RE			SEM	
			
	24 h	48 h	72 h	24 h	48 h	72 h	24 h	48 h	72 h	day 10	
Daily											
Mesor (°C)	39.22^e^	39.27^e^	39.36^d^	39.49^c^	40.09^b^	40.48^a^	39.03^f^	39.07^f^	38.93^g^	39.26^e^	0.03
Zenith (°C)	39.68	39.61	39.69	40.17	41.06	41.15	39.60	39.48	39.18	39.59	-
Acrophase (h)	19:20	19:40	19:50	19:30	19:10	12:40	16:30	21:20	19:40	19:20	-
Nadir (°C)	38.83	38.87	38.98	38.66	39.20	39.15	38.31	38.64	38.66	38.77	-
Trough (h)	05:30	06:30	06:00	06:30	05:40	06:40	08:30	06:20	06:20	06:00	-
Range of oscillation (°C)	0.85	0.74	0.70	1.51	1.86	2.00	1.29	0.84	0.52	0.82	-
Stage											
Mesor (°C)		39.18^b^			40.09^a^			39.01^c^		39.15^b^	0.02
Zenith (°C)		39.62			40.73			39.25		39.59	-
Acrophase (h)		19:40			19:10			20:10		19:20	-
Nadir (°C)		38.90			39.08			38.62		38.77	-
Trough (h)		06:30			06:30			06:20		06:00	-
Range of oscillation (°C)		0.72			1.65			0.63		0.82	-

SEM, standard error of the mean.

1) Refer to the accompanying text for further elucidation.

2) EU, euhydration stage; DE, dehydration stage; RE, rehydration stage; RE_10_, day 10 of the rehydration stage.

a–g Means bearing different superscripts are significantly different at p<0.05.
